# The Role of the Enteral Route and the Composition of Feeds in the Nutritional Support of Malnourished Surgical Patients

**DOI:** 10.3390/nu4091230

**Published:** 2012-09-04

**Authors:** Luca Nespoli, Sara Coppola, Luca Gianotti

**Affiliations:** Department of Surgery, Milano-Bicocca University, San Gerardo Hospital, Monza, 20900, Italy; Email: luca.nespoli@unimib.it (L.N.); sara-cop@libero.it (S.C.)

**Keywords:** malnutrition, enteral nutrition, immunonutrition, surgery

## Abstract

In surgical patients, malnutrition is an important risk factor for post-operative complications. In undernourished patients undergoing major gastrointestinal procedures, preoperative enteral nutrition (EN) should be preferred whenever feasible. It may be given either orally or by feeding tubes, depending on patient compliance. Early oral intake after surgery should be encouraged, but if an insufficient postoperative oral intake is anticipated, tube feeding should be initiated as soon as possible. The use of immunomodulating formulas offers significant advantages when compared to standard feeds and the positive results on postoperative complications seem independent from the baseline nutritional status. In malnourished patients, the optimal timing and dose of immunonutrition is unclear, but consistent data suggest that they should be treated peri-operatively for at least two weeks.

## 1. Introduction

Surgical trauma, as with any other injury, is known to affect the host metabolism, immune defense mechanisms and inflammatory response [1,2]. Such alterations of the homeostasis may lead to impaired tissue healing and organ function and eventually to a poor outcome. 

Malnutrition, as defined in Table 1 [3,4,5,6] has been recognized as an independent risk factor for increased post-operative morbidity and mortality, prolonged length of hospital stay and increased costs [3,7]. Major surgical procedures are often performed in tumor-bearing patients. Their nutritional status may be even more compromised for the development of tumor cachexia.

**Table 1:** Definition of malnutrition. Common screening tools for malnutrition.

Malnutrition	Weight loss	BMI (kg/m^2^)	SGA	Albumin (g/L)	NRI [3]	NRS [4]
**None**	-	18.5–25	A	>35	>97.5	Score 0
**Mild**	<10%	<18.5	B	<35	84–97.5	Score 1
**Moderate**						Score 2
**Severe**	>10%	<16	C	<30	<84	Score 3

According to the European Society for Enteral and Parenteral Nutrition (ESPEN) criteria [5,6]; BMI: Body mass index; SGA: subjective global assessment; NRI: nutritional risk index; NRS: nutritional risk screening.

When major surgical complications occur, the subsequent overwhelming catabolic and inflammatory responses may further worsen the healing host ability and organ function. It has been shown that inadequate calorie and nitrogen intake for more than 14 days and severe catabolism significantly increase surgery-related morbidity and mortality [8]. Therefore, it has been hypothesized that a tailored nutritional support may play a key role in counterbalancing the detrimental effects of both pre-existing malnutrition and surgical stress.

## 2. Pre-Operative Energy and Nitrogen Support

In patients with cancer of the gastrointestinal tract, severe malnutrition often reflects an advanced-stage disease not suitable for radical surgical therapy. Yet, in those patients in whom a radical oncologic surgery is feasible, the impact of pre-operative nutritional support by total parenteral nutrition (TPN) on clinical outcome has been addressed in several trials [9,10,11]. The results are controversial, but international guidelines [5,6] suggest that the administration of 10–15 days of TPN in the pre-operative period may improve postoperative morbidity. This beneficial effect has been clearly established only in severely malnourished patients. 

The implementation of such guidelines may be difficult in clinical practice. In fact, pre-operative TPN requires management in hospital settings: prolonging length of stay and increasing the risk of nosocomial infections and costs. 

In the presence of a functioning gut, preoperative enteral nutrition (EN), by means of oral nutritional supplements (ONS) or tube feeding, may be performed safely as outpatient therapy and offers several advantages over TPN. The use of EN may achieve prevention of bowel mucosal atrophy, enhancement of local blood perfusion and local immune response, promotion of enterocyte turnover, preservation of the barrier function against microbial translocation and reduction of sanitary costs [12,13].

Hypercaloric enteral tube feeding for 10 days before surgery was shown to reduce morbidity and mortality, as well as length of stay, in malnourished surgical patients, when compared with normal feeding [14] or TPN [15].

### Pre-Operative Immuno-Metabolic Support

Although the above concepts and indications of classic artificial nutrition remain valid, extensive clinical research in the last 20 years has clearly shown that the administration of supernormal doses of specific nutritional substrates may have immuno-modulatory, anti-inflammatory, anabolic, and tissue protective effects. This translates into improved surgical outcome when compared with standard nutritional formulas or traditional treatment protocols. For this reason, this new area of nutritional therapy has been generically named immunonutrition (IMN) or pharmaconutrition. Immune-enhancing formulas are usually enriched with various combinations and doses of arginine, glutamine, nucleotides, and omega-3 fatty acids, which can be administered both orally and through feeding tubes. Their mechanisms of action have been reviewed by others [16,17,18]. 

An effective modulation of host response requires the attainment of adequate plasma and tissue levels of these substrates during the first postoperative days to effectively counterbalance the detrimental effects of postsurgical inflammation and immunosuppression. In such a perspective, pre-operative IMN might represent an ideal approach by achieving a substrate load before operation. Indeed, the amount of substrates that are provided in the immediate postsurgical period might be insufficient because of the limited amount of enteral feeding tolerated in the first days of treatment. Nevertheless, the immuno-metabolic activity of these specific nutrients is largely proven [19,20,21,22,23,24,25].

Three recent meta-analyses [26,27,28] studied the effect of IMN on surgical-related outcome measures. All of them consistently showed that when IMN was given pre-operatively, it was able to reduce significantly the overall complication rate and the length of hospitalization. One of the limitations of the above meta-analyses was the lack of stratification by the pre-operative nutritional status. In most of the studies analyzed, enrolled subjects had no malnutrition or a mild weight loss.

## 3. Post-Operative Energy and Nitrogen Support

In modern surgical practice, most surgical patients can be managed according to an enhanced recovery protocol [29], allowing them to receive oral feeding within a few days after an operation. Therefore, only those patients at high risk of postoperative complications who cannot be fed adequately by the oral route within 7–10 days should be treated by post-operative artificial nutrition. Contrariwise, patients with pre-operative severe under-nutrition should be treated with artificial nutrition without delay if an inadequate oral intake for more than 7 days postoperatively is anticipated [5,6]. In the absence of contraindications (intestinal obstructions, ileus, severe shock, intestinal ischemia), the enteral route of administration should be preferred. Meta-analyses pooled the results of several randomized trials, comparing EN to TPN [30,31]. The enterally-fed patients had significantly less post-operative infections and shorter hospital stays.

When planning or performing early post-operative EN, adverse events and related complication should be taken into account. In a revision of 650 patients treated by early EN after major digestive surgery for cancer [32], we observed minor side effects (abdominal cramps, bloating, diarrhea, and vomiting) in about 30% of patients. Most of these events were successfully managed by symptomatic medications or by reducing the infusion rate. The nutritional goal was achieved within the fourth postoperative day in 82% of the patients, and 91% of the patients reached the nutritional goal between the fourth and seventh days. A definitive switch to TPN was needed in the remaining 9% of patients.

The overall complication rate related to jejunostomy or to a naso-jejunal tube was 6.1%. Tube dislodgement and clogging were the most commonly observed complications and they were more frequent in patients having naso-jejunal tubes. The re-intervention rate was 1.7% in patients with a jejunostomy tube, due to dislodgment or intestinal obstruction.

### Post-Operative Immuno-Metabolic Support

The effects of immune-enhanced formulas in the postoperative period on primary outcome measures have also been investigated by several meta-analyses [26,27,28]. The conclusions were quite similar, despite the differences in study selection. All of them clearly showed a significant reduction of about 50% of postoperative infectious complication rate and of 2 days’ length of hospital stay (LOS) in the groups receiving IMN, when compared to standard feeds. Again, none of the above meta-analysis stratified trials accorded with the baseline nutritional status and risk. This may be of paramount importance to understand which subgroup of patients can benefit most from IMN and its ideal timing of administration.

Five RCTs and one case series addressed the effect of IMN selectively in malnourished patients (Table 2). The heterogeneity of the study design, the distinctive type of formulas used, and the different periods of feeding make it difficult to pool data and draw any definitive conclusions. Nevertheless, from the results reported, it appears that in malnourished patients, the optimal approach is to start IMN before surgery for at least 7 days and prolong after surgery for the same period of time. This may be achieved by oral administration or by feeding tubes, depending on patient compliance. 

**Table 2:** Studies evaluating the effect of IMN in malnourished patients.

	Braga 1998 [33]	Braga 1999 [34]	Braga 2002 [35]	Bozzetti 2007 [7]	Klek 2011 [36]	Klek 2011 [37]
Type of study	RCT	RCT	RCT	Case series	RCT	RCT
Sample size	266	206	150	1410	167	305
Type of analysis	*Post-hoc* (*n* = 78 malnourished)	*Post-hoc* (*n* = 40 malnourished)	ITT	*Post-hoc* (*n* = 806 malnourished)	ITT	ITT
Definition of malnutrition	WL > 10%	WL > 10%	WL > 10%	WL > 10%	ESPEN criteria	WL > 10% or BMI < 18
IMN formula	Impact^®^	Impact^®^	Impact^®^	Impact^®^	Stresson^®^	Reconvan^®^
Type of control group	Standard enteral diet, TPN	Standard enteral diet	Standard enteral diet	TPN, I.V. glucose, standard enteral diet	Standard enteral diet	Standard enteral diet
Timing of IMN	Post-op	Peri-op	Peri-op	Pre-, peri- , and post-op	Post-op	Post-op
Duration of IMN	Until adequate oral feeding	14 days	14 days	Variable	7 days	7 days
Improved outcome	Yes	Yes	Yes	Yes	No	Yes

RCT: randomized clinical trial; ITT: intention-to-treat; WL: weight loss.

## 4. Conclusions

Malnutrition is an important risk factor for post-operative complications in surgical patients. In undernourished patients undergoing major gastrointestinal procedures, pre-operative EN should be preferred whenever feasible. Early oral feeding after surgery should be encouraged, but when an insufficient post-operative oral intake is anticipated, tube feeding should be initiated at once. The use of immunomodulating formulas offers significant advantages when compared to standard feeds, and the positive results on primary outcomes seem independent from the baseline nutritional status. In malnourished patients, the optimal timing and dose of IMN is unclear, but consistent data suggest that they should be treated peri-operatively for at least two weeks. 
